# High-grade serous papillary ovarian carcinoma combined with nonkeratinizing squamous cell carcinoma of the cervix: a case report

**DOI:** 10.3389/fonc.2024.1298109

**Published:** 2024-03-07

**Authors:** Maoyuan Wu, Wenwen Zhang, Lianli He, Ye Zhu, Xiaoling Jiang, Lixia Zhang, Xiwei Yuan, Tingchao Li

**Affiliations:** ^1^ Department of Gynecology and Obstetrics, The Third Affiliated Hospital of Zunyi Medical University (The First People’s Hospital of Zunyi), Zunyi, Guizhou, China; ^2^ Department of Gynecology, The Third Affiliated Hospital of Zunyi Medical University (The First People’s Hospital of Zunyi), Zunyi, Guizhou, China; ^3^ Department of Imaging, The Third Affiliated Hospital of Zunyi Medical University (The First People’s Hospital of Zunyi), Zunyi, China; ^4^ Department of Pathology, The Third Affiliated Hospital of Zunyi Medical University (The First People’s Hospital of Zunyi), Zunyi, China

**Keywords:** case report, multiple primary malignant neoplasms, cervical cancer, ovarian cancer, high-grade serous papillary carcinoma

## Abstract

Multiple primary malignant neoplasms are a rare gynecologic malignancy; particularly, cases originating from the heterologous organs, such as the ovary and cervix. Here, we report a case of two primary malignant neoplasms in a patient who had undergone laparoscopic radical hysterectomy + bilateral salpingo-oophorectomy + pelvic lymph node dissection + para-aortic lymphadenectomy + appendectomy + omentectomy + metastasectomy under general anesthesia. The patient experienced complete remission after six courses of postoperative chemotherapy with a standard Taxol and Carboplatin regimen. Genetic testing was performed to detect *BRCA2* mutations, and poly (ADP-ribose) polymerase (PARP) inhibitors were used for maintenance therapy.

## 1 Introduction

Multiple primary malignant neoplasms (MPMN) were first described by Billroth et al. in 1889 (1). Many clinical cases of MPMN have been reported since. Most patients have metachronous MPMN as MPMN occurrences are rarely synchronous. MPMNs originating from the female reproductive system account for 1–2% of the cases of gynecologic malignant neoplasms. Among these cases, the most prevalent ones, accounting for approximately 50–70% of cases, have synchronous ovarian and endometrial cancers arising from homologous tissues (2, 3). However, cases of malignant neoplasms synchronously arising from the cervix and ovary are extremely rare.

Here, we report a case of high-grade serous papillary carcinoma (HGSOC) of the ovary combined with nonkeratinizing squamous cell carcinoma (SCC) of the cervix with multiple metastases from the HGSOC.

## 2 Case report

**Figure 1:**
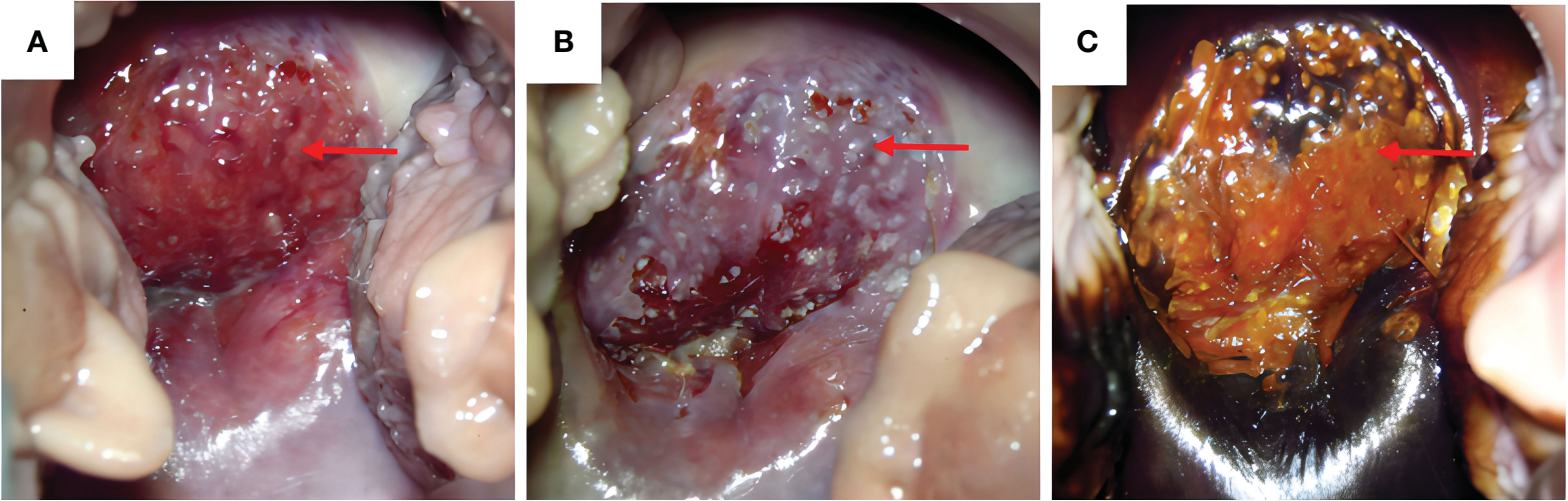
Morphology of cervical lesions. **(A)** Colposcopy examination of the cervical lesions. Red arrows indicate the cervical lesions. **(B)** Vinegar test: opaque white spots with the white color fading slowly and presence of mosaic blood vessels. **(C)** Cervical iodine test result: no change in color in the iodine test of the anterior cervical lip.

**Figure 2:**
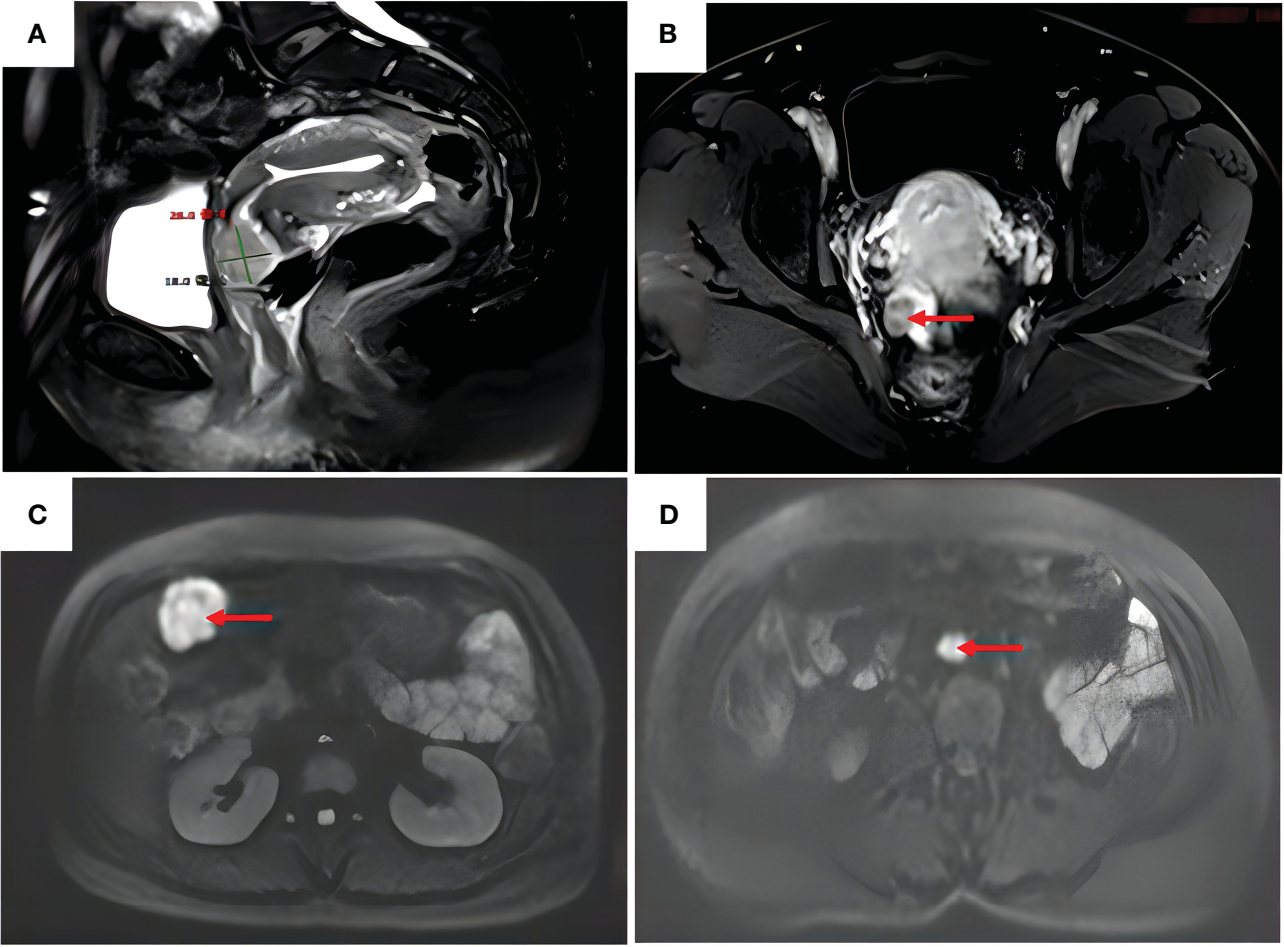
Full abdomen + pelvis magnetic resonance imaging. **(A)** Anterior cervical lip with a slightly enhanced signal on T2–weighted images, approximately 26 mm in diameter. **(B)** Enhancement of the right pelvic nodal lesion (red arrow) on T1–weighted images. **(C)** Enhanced signal of the mass in the right upper abdomen (red arrow) on diffusion–weighted images. **(D)** Enlarged retroperitoneal abdominal para–aortic lymph nodes (red arrow) on diffusion–weighted images.

**Figure 3:**
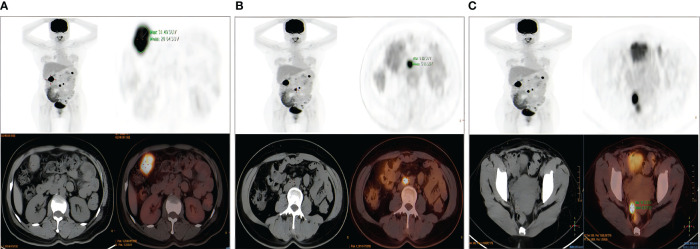
Positron emission tomography–computed tomography. **(A)** Nodular lesion anterior to the hepatic flexure of colon. **(B)** Enlarged retroperitoneal abdominal para–aortic lymph nodes. **(C)** Multiple soft–tissue masses and nodules on the right wall of the pelvis.

A 47-year-old woman was admitted to the hospital because of irregular vaginal bleeding for more than one month. She had family history of a first-degree relative dying from a brain tumor, two second-degree relatives dying from lung cancer, and another relative dying from a gynecologic malignancy. The ancillary test results suggested the presence of human papillomavirus (HPV) 16 and atypical SCC cells in the ThinPrep cytologic test; therefore, the possibility of high-grade squamous intraepithelial lesions could not be excluded. The results of the cervical biopsy suggested grade-III cervical intraepithelial neoplasia in the cervical canal. Cervical infiltration localized to grade-III cervical intraepithelial neoplasia was suspected, and the possibility of SCC could not be excluded. Colposcopy revealed erosion-like changes in the anterior cervical lip, vascular proliferation, and bleeding after contact (**Figure 1A**). The vinegar test revealed areas that turned opaque white with the white stain fading slowly. Mosaic blood vessels were observed (**Figure 1B**), with no change in color in the iodine test (**Figure 1C**). Full abdominal + pelvic magnetic resonance imaging showed a cervical mass of approximately 2.6 cm in the maximum diameter (**Figure 2A**); a nodular mass on the right side of the pelvis, which was considered a metastatic lesion (**Figure 2B**); multiple space-occupying lesions in the abdominal cavity (**Figure 2C**); and enlarged retroperitoneal lymph nodes (**Figure 2D**), also considered to be metastatic lesions. Positron emission tomography-computed tomography showed space-occupying lesions in the cervix; multiple soft-tissue masses and nodules anterior to the hepatic flexure (**Figure 3A**), adjacent to the greater curvature of the stomach, retroperitoneal and adjacent to the abdominal aorta (**Figure 3B**), and on the right wall of the pelvis (**Figure 3C**); and abnormally elevated glucose levels. Laboratory tests revealed a CA125 level of 45.8 U/mL and an epithelial SCC antigen level of 4.2 ng/mL.

**Figure 4:**
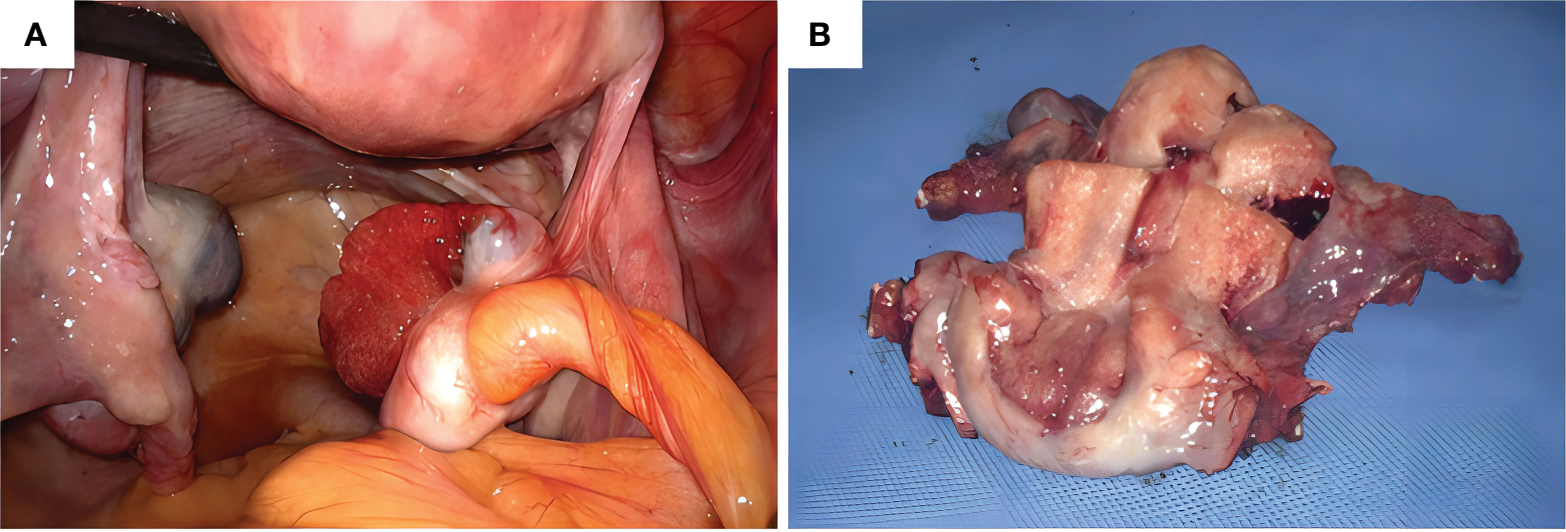
**(A)** Intraoperative observation of a neoplasm of approximately 3.0 cm in size in the right ovary, **(B)** Uterine specimen: The tumor is found in the cervical canal, with a diameter of 2+ cm and a nodular shape.

**Figure 5:**
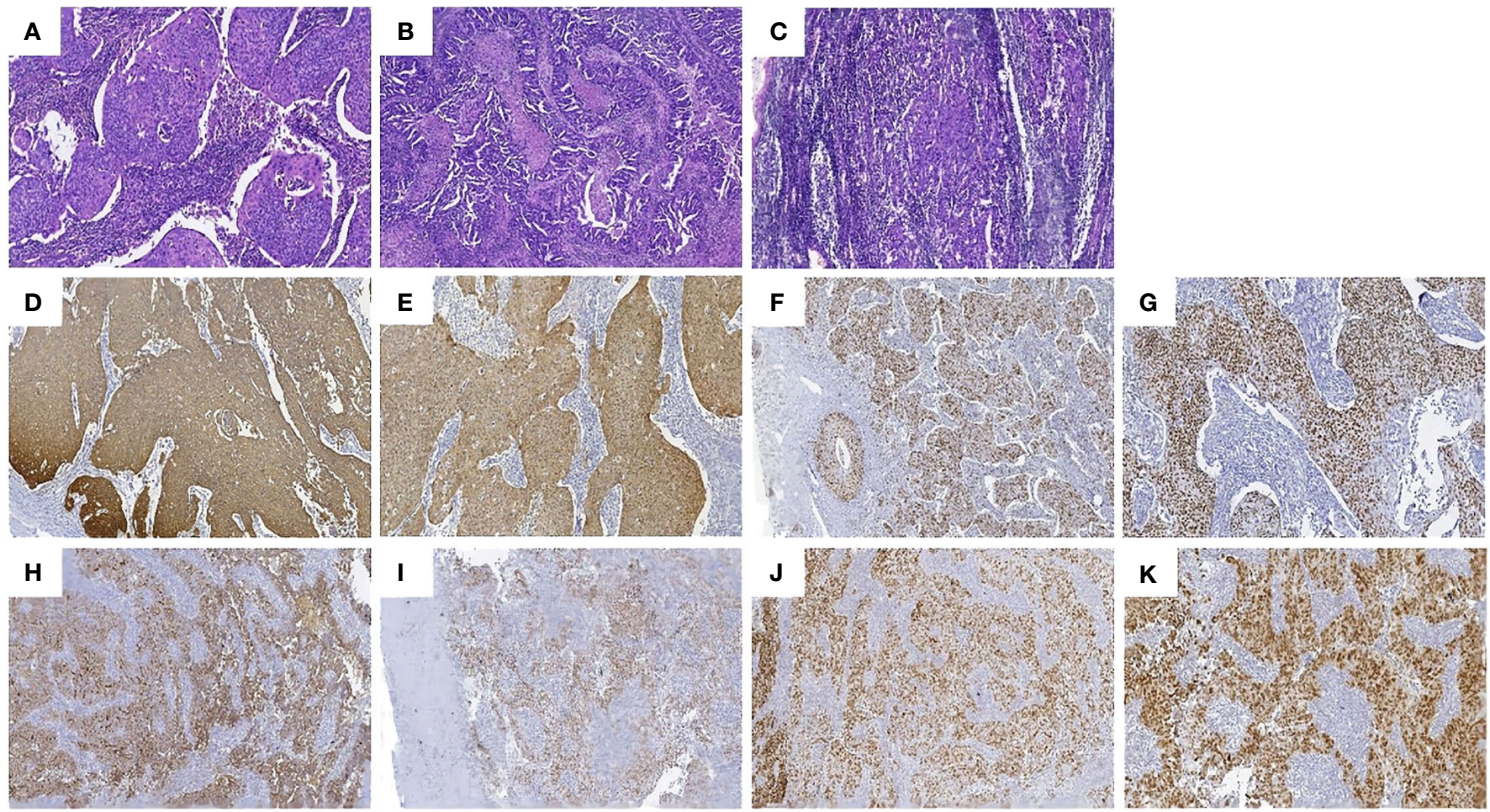
Pathologic findings: **(A)** Hematoxylin and eosin **(HE)** staining of the nonkeratinizing SCC of the cervix. **(B)** Hematoxylin and eosin staining of the HGSOC. **(C)** hematoxylin and eosin staining of the enlarged lymph nodes adjacent to the abdominal aorta. The SCC area had a nest–like distribution of the tumor cells, patchy necrosis, pathological karyorrhexis, and invasive growth. Immunohistochemistry: tumor cells in the SCC of the cervix were diffusely positive for **(D)** CK5/6, **(E)** p16, **(F)** Ki–67, and **(G)** p40, and the tumor cells in the HGSOC were diffusely positive for **(H)** CA125, **(I)** WT–1, **(J)** Pax–8, and **(K)** p53.

We obtained informed consent from patients and families for all of our treatments. On June 6, 2023, the patient underwent laparoscopic surgery. Intraoperatively, the right ovary showed a neoplasm of approximately 3.0 cm (**Figure 4A**). Two enlarged lymph nodes were spotted in the upper abdomen next to the abdominal aorta, and the size of the omental metastatic mass was approximately 5.0 cm. Therefore, under laparoscopic guidance, we performed radical hysterectomy + bilateral salpingo-oophorectomy + pelvic lymph node dissection + para-aortic lymphadenectomy + appendectomy + omentectomy + metastasectomy and administrated 100 mg of cisplatin intraperitoneally for chemotherapy. Postoperative dissection of the uterine specimen revealed a tumor approximately 2+ cm in diameter and nodular in shape located in the cervical canal (**Figure 4B**). Pathological examination confirmed that the cervical mass was nonkeratinizing SCC (**Figure 5A**), with the tumor infiltrating nearly the entire layer (> three-fourths) and cervical canal and lympho-vascular and perineural invasions. Furthermore, the right ovarian neoplasm was HGSOC (**Figure 5B**). The patient had a para-abdominal aortic lymph node metastasis, with no metastasis in the 21 left or 15 right pelvic lymph nodes. The two para-abdominal aortic lymph nodes had metastasis, and two cancerous masses were found in the omentum. Immunohistochemical examinations showed CK5/6 (+; **Figure 5C**), p16 (+; **Figure 5D**), Ki-67 (+; approximately 80%; **Figure 5E**), and p40 (+; **Figure 5F**) in the cervical tumor tissue. Ovarian cancer tissue was CA125 (+; **Figure 5G**), WT-1 (+), Pax-8 (+; **Figure 5H**), p53 (strongly diffused +; **Figure 5I**), p16 (+), CK7 (partially +), CD1 (–), CK2O (–, **Figure 5J**), CDX–2 (–), and Ki–67 (+, approximately 80%). Postoperative diagnoses were stage IIIC2 HGSOC and stage IB2 nonkeratinizing SCC of the cervix.

**Figure 6:**
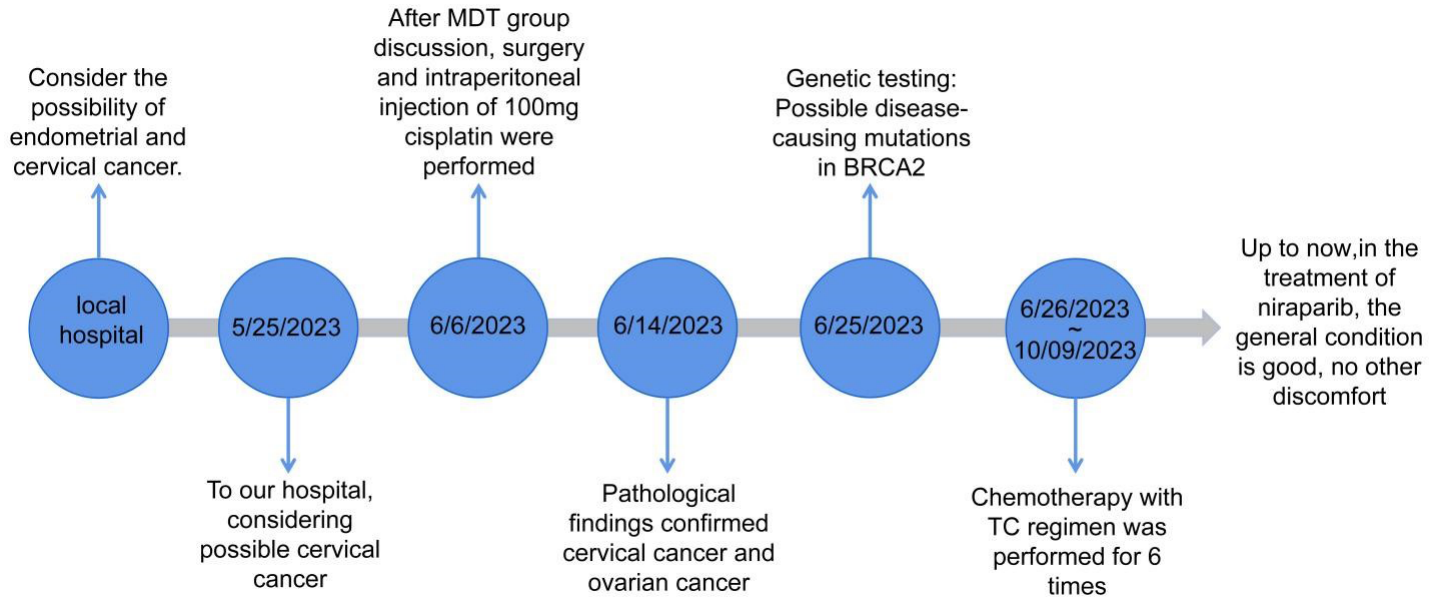
Timeline of the patient.

As the patient had nonkeratinizing SCC of the cervix, with the tumor infiltrating nearly the entire layer (> three–fourths) and cervical canal and lympho–vascular and perineural invasions, which were considered high–risk factors, the patient was advised to receive simultaneous radiotherapy. However, she refused to undergo radiotherapy, and six courses of chemotherapy with a standard docetaxel and cyclophosphamide regimen were administrated instead. Genetic testing revealed a possible pathogenic mutation on BRCA2p.K503Sfs*6 with a homologous recombination deficiency (HRD) score of 64. Since the HRD score was > 42, the patient was assessed to be HRD–positive after a comprehensive evaluation. The patient may respond well to the treatment with poly (ADP–ribose) polymerase inhibitors, such as Olaparib and Niraparib, and platinum–based drugs. In October 2023, after the completion of chemotherapy patients began to oral Niraparib. The timeline of the patient’s entire treatment process was depicted in **Figure 6**.

## 3 Discussion

MPMN is the simultaneous or sequential occurrences of two or more histologically unrelated primary malignant lesions in the same individual. MPMN, or multiple carcinomas and multiple primary carcinomas, can originate from the same organ, paired organs, different parts of the same system, or different organs of different systems. This concept was first described in 1989. (4–6) MPMN is rare in clinical practice, accounting for only 0.52–11.7% of all malignant neoplasms (7). Based on the time interval between the occurrence of different primary tumors, MPMN can be further classified into (1) synchronous: relatively rare cases in which the intervals between the occurrences of MPMN are < 6 months, and (2) metachronous: common cases in which the intervals between the occurrences of MPMN are ≥ 6 months, (8) accounting for approximately 90% of MPMN cases. The diagnostic criteria for MPMN are (1): each tumor confirmed as malignant based on pathological findings; (2) each tumor with distinct pathomorphological features; (3) tumors occurring at different sites or organs or different parts of the same organ and not in contact with each other; (4) each tumor with its distinct metastatic modes and routes, and (5) cases of tumor recurrence and metastasis excluded (9). MPMNs originating from the female reproductive system are relatively rare.

In this case report, the patient was successively diagnosed with cervical non–keratinizing squamous cell carcinoma and ovarian high–grade serous papillary carcinoma within one month. The pathological type and primary site of this case were different, which met the diagnostic criteria for synchronous MPMN. Among synchronous malignant neoplasms of the female reproductive tract, synchronous endometrial and ovarian cancer is the most common, accounting for 50–70% of the cases, with the pathologic type being low–grade endometrioid carcinoma (10). Unlike ovarian cancers, cervical cancers originate in the cervix, most of which are SCCs. A case of synchronously occurring cervical SCC and ovarian cancer such as this is very rare.

**Figure 7:**
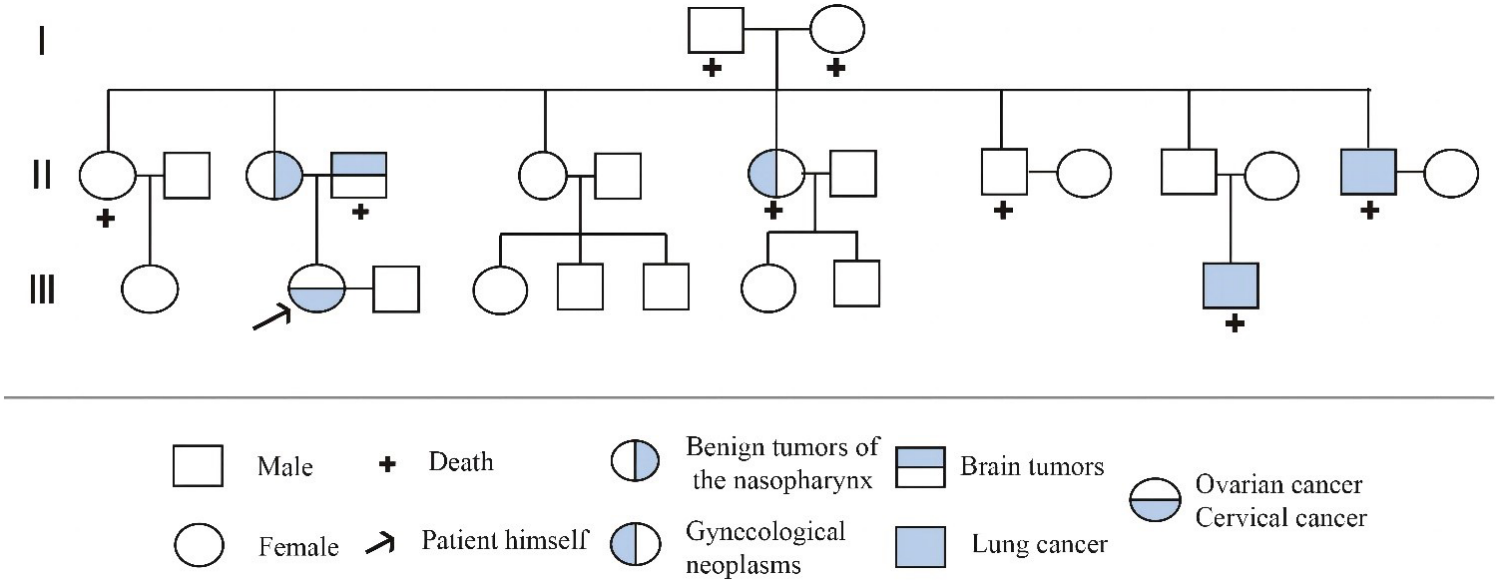
Pedigree of the family. the circle (○) represents the female, the square (□) represents the male, “+” represents the death, the arrow represents the patient himself, and the Roman numeral represents the generation. Among the patient’s family members,one person died due to brain tumor, one died due to gynecological tumor, and two died due to lung cancer.

Factors affecting the pathogenesis of MPMN remain unclear but are generally believed to be related to genetic and carcinogenic factors, host susceptibility, carcinogenic side effects of radiotherapy, immunodeficiency, and unhealthy lifestyles. (11, 12) Therefore, some patients have increased cancer susceptibility (13). We carefully obtained the medical history and history of exposure to toxic or hazardous substances. The family history showed that a first–degree relative had died from a brain tumor, two second–degree relatives had died from lung cancer, and another second–degree relative had died from a gynecologic malignancy (**Figure 7**). Although these relatives did not undergo genetic testing, breast cancer gene 2 (*BRCA2*) has been shown to be under–expressed in the tissues of six cases of lung SCC. *BRCA2* is considered to be a biomarker for lung SCC, (14) leading to the speculation that *BRCA2* mutations are associated with the onsets of lung cancers in these second–degree relatives of the patient. In this case report, the patient had an abnormal genetic test result showing a *BRCA2* mutation and was considered to have a possible family history of this mutation.

*BRCA1* and *2* are tumor–suppressor genes that play important roles in processes such as normal cell growth and DNA damage repair. If the tumor suppressor genes are inactivated because of mutation, deletion, or loss of expression, they cannot restrict tissue growth. More than 100 discrete germlines of *BRCA1* and *2* mutations have been identified, *BRCA* mutations penetrating the germlines during the human lifetime is very high (15). Hereditary breast and ovarian cancers caused by *BRCA1* and *2* mutations are inherited in an autosomal dominant pattern and account for approximately half of the cancer cases associated with hereditary risks. Approximately 80% of hereditary ovarian cancers are attributed to *BRCA* mutations (16).

Data from the National Cancer Institute Surveillance, Epidemiology and End Results Program (NCI SEER) reported that the ovarian cancer with 19880 new cases in 2022 and a 5–year relative survival of 49.7%, cervical cancer with 14100 new cases in 2022 and a 5–year relative survival of 66.7% (17). Ovarian cancer is a common cause of mortality related to gynecologic cancer. Due to the lack of obvious early clinical symptoms and effective screening methods, approximately 70% of patients with epithelial ovarian cancer in the clinic practice are in the advanced stage of the disease at the time of diagnosis (18). Approximately 20 genes are associated with ovarian cancer, with *BRCA* having the most significant impact. *BRCA2* mutations increase the risk of ovarian cancer by approximately 11.4% (19). Other cancers associated with *BRCA2* mutations include pancreatic cancer, prostate cancer, stomach cancer, cholangiocarcinoma, gallbladder cancer, and melanoma.

This patient also had a high–risk HPV 16 infection. Persistent HPV infection is a major risk factor for cervical cancer (20). Postoperative immunohistochemistry examination of the cervical cancer tissues showed positive P16 staining, indicating that persistent HPV infection led to the alteration in cell physiology. Thus, the infection was a probable cause of cervical cancer in this patient. As a tumor–suppressor gene, the p16 expression is reduced in primary tumors. However, p16 is highly expressed in cervical cancer and high–grade precancerous cervical lesions. A study showed that the application of a screening protocol for cervical lesions constituting an HPV test combined with a ThinPrep cytologic test for p16 enables an early diagnosis and, therefore, timely treatment (21).

The onset and progression of HGSOC are highly insidious, and HGSOC is the deadliest gynecologic malignancy (22). The serum CA125 level is the most used biomarker in ovarian cancer screening, and the sensitivity and specificity for differentiating malignant from benign tumors in postmenopausal women with abdominal masses range from 50–100%. However, it has limited clinical application and is incapable of identifying early–stage ovarian cancer. This is because the elevated CA125 level can be due to inflammations in diseases such as endometriosis, pelvic inflammatory disease, tuberculous peritonitis, and pancreatitis (23). Additionally, other biomarkers, including autoantibodies, ctDNA, methylation, and miRNA, have potential for early detection of ovarian cancer. More attention should be paid to these biomarkers as well as their clinical applications (24). Of all female patients, 50% early–stage ovarian cancer do not have elevated CA125 levels. Therefore, most female patients are at stage III or IV at the time of diagnosis since they only seek medical help when they experience symptoms of distant metastases.

This patient, who had undergone surgery for cervical cancer, developed multiple metastases suggested by the preoperative magnetic resonance imaging and positron emission tomography–computed tomography, including metastases to the lymph nodes adjacent to the abdominal aorta, reaching as far as the site adjacent to the greater curvature of the stomach. Moreover, the nodular mass on the right wall of the pelvis was considered a metastatic lesion. However, the most common routes for cervical carcinoma to metastasize are through direct spread and lymph nodes. Both routes of metastasis through lymph nodes end up in the para–abdominal aortic lymph nodes. The chance of the metastasis reaching the para–abdominal aortic area was low, this is mostly seen in advanced invasive cervical cancer (25). Therefore, we considered the possibility of the presence of other primary malignancies in this patient and performed an abdominal exploration. As predicted, a 3–cm neoplasm was found intraoperatively on the right ovary, and the findings of the cryosection pathological examination suggested malignancy. Thus, radical hysterectomy and debulking cytoreductive surgery were performed. The results of the postoperative pathological examinations suggested that the right ovarian mass was HGSOC, and the results of the hematoxylin and eosin staining demonstrated that the multiple metastatic lesions were all serous papillary carcinomas of an ovarian origin. According to the 2013 International Federation of Gynecology and Obstetrics staging, the patient had stage IIIC2 ovarian cancer (26). The cervical carcinoma did not metastasize and was classified as stage IB2 cervical cancer according to the 2013 International Federation of Gynecology and Obstetrics classification (27). High–risk factors, such as tumor infiltration in nearly the entire layer (> three–fourths), cervical canal, and lympho–vascular and perineural invasions, were observed in this patient. Therefore, radiotherapy was recommended based on the National Comprehensive Cancer Network guidelines. However, the evidence level was low (28). The rate of abdominal metastasis from ovarian cancer was as high as 70%. Adjuvant intraperitoneal chemotherapy after a satisfactory debulking surgery has a better survival benefit compared to intravenous chemotherapy in patients with advanced ovarian cancer (29). Therefore, we administered 100 mg of cisplatin for intraperitoneal perfusion chemotherapy.Through abdominal cavity perfusion of cisplatin, can kill and eliminate the intraperitoneal free cancer cells, reduce transfer rate and postoperative recurrence rate. At the same time, because the drug does not directly enter the human circulation, the damage to the kidney is less, and the incidence of gastrointestinal adverse reactions is low (30).

The onset of HGSOC in this patient is considered to be strongly associated with the *BRCA2* mutation. Maintenance treatment with Niraparib was implemented after six courses of chemotherapy with a standard docetaxel and cyclophosphamide regimen (31). Poly–ADP ribose polymerase inhibitors selectively kill tumor cells with HRD due to mutations in *BRCA* but do not affect cells with normal *BRCA* genes (32). The therapeutic efficacy of poly–ADP ribose polymerase inhibitors has been demonstrated in patients with advanced ovarian cancer with *BRCA1/2* mutations (33). In addition, Olaparib can reduce the risk of disease progression or death by 70% compared to placebo. However, the increasing drug resistance in patients should be addressed. The patient has three daughters, the youngest of whom is currently in elementary school. Considering the patient, as a mother, has a known *BRCA2* mutation with a current diagnosis of ovarian cancer and a family history of cancer, genetic tests were recommended for all of her daughters. If their results are also suggestive of the same mutation, risk–reducing salpingo–oophorectomy is recommended as the standard risk–reducing procedure according to the National Comprehensive Cancer Network guidelines (34). Risk–reducing salpingo–oophorectomy reduces the risk of breast cancer in carriers of *BRCA1/2* mutations, but the magnitude of impact remains unknown (35).

## 4 Conclusions

Clinicians should improve their understanding and awareness of MPMN to reduce the rates of misdiagnosis and underdiagnosis. Clinicians should clarify whether tumors in multiple organs are recurrences, metastases, or primary growths of malignant neoplasms, which is of far–reaching significance in the diagnosis and treatment of this disease type. The cancer type in this case is rare. Therefore, treatment strategies should be developed in a highly customized manner for individual patients to maximize therapeutic benefit. A close multidisciplinary collaboration is particularly important to effectively treat and manage this disease type. Additionally, patients should be encouraged to lead a healthy lifestyle by, for instance, exercising appropriately, balancing work and rest time, avoiding excessive fat intake, and abstaining from smoking and alcohol intake. Finally, we should emphasize the patient follow–ups, which can help to timely detect the recurrence and metastasis of the first primary cancer and other primary cancers, thereby ultimately improving the patients prognosis.

## Data availability statement

The original contributions presented in the study are included in the article/**Supplementary Material**. Further inquiries can be directed to the corresponding author.

## Ethics statement

Ethical review and approval was not required for the study on human participants in accordance with the local legislation and institutional requirements. Written informed consent from the patients/participants or patients/participants’ legal guardian/next of kin was not required to participate in this study in accordance with the national legislation and the institutional requirements. Written informed consent was obtained from the individual(s) for the publication of any potentially identifiable images or data included in this article.

## Author contributions

MW: Investigation, Writing – original draft, Resources. WZ: Writing – original draft. LH: Writing – review & editing. YZ: Writing – review & editing. XJ: Writing – review & editing. LZ: Writing – review & editing. XY: Writing – review & editing. TL: Writing – review & editing.
