# Supplementary material for: High-grade serous papillary ovarian carcinoma combined with nonkeratinizing squamous cell carcinoma of the cervix: a case report
Source: Front Oncol. 2024 Mar 7;14:1298109. doi: 10.3389/fonc.2024.1298109 (PMC10956574; doi:10.3389/fonc.2024.1298109)

Supplementary Material

Supplementary Figures

Supplementary Figure 1

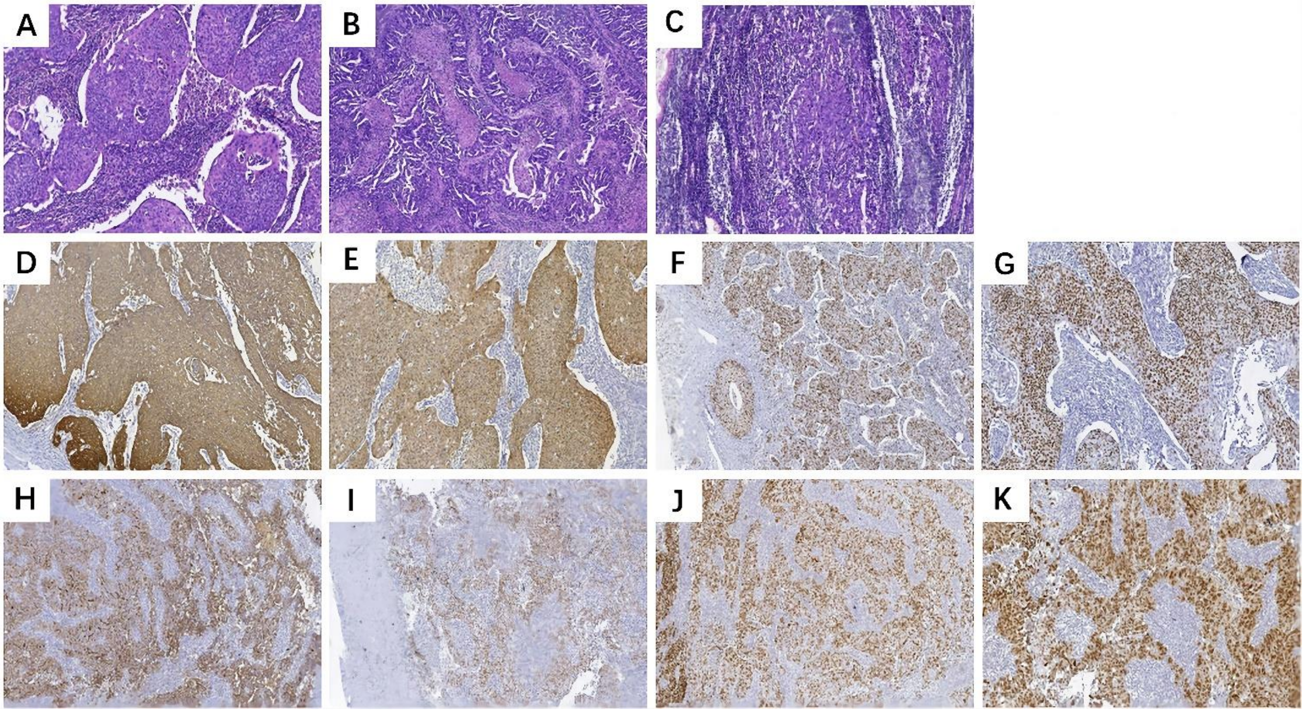

Supplementary Figure 2

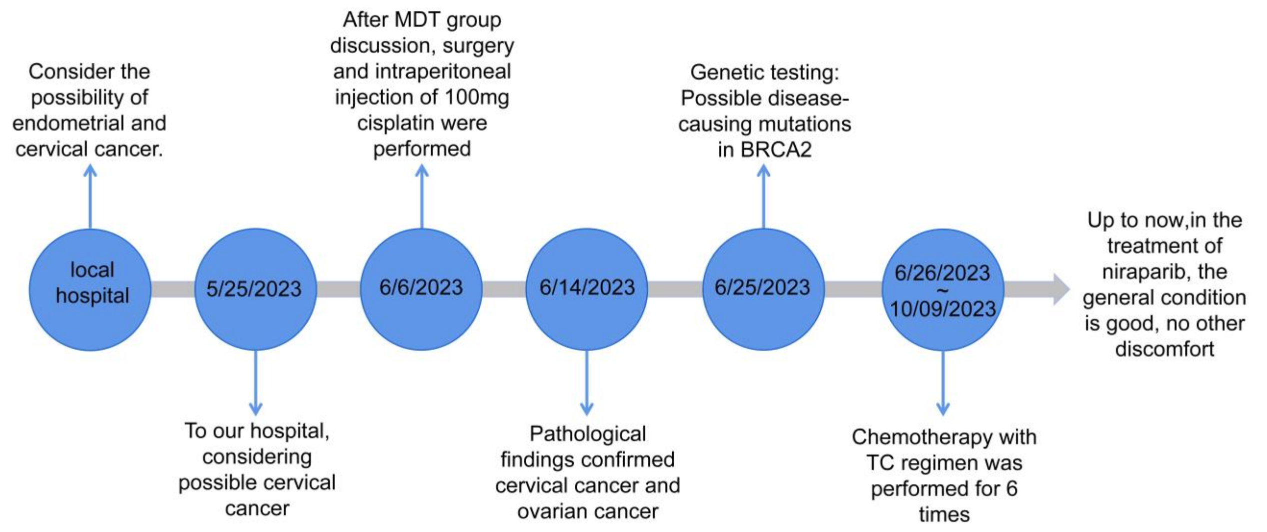

Supplementary Figure 3

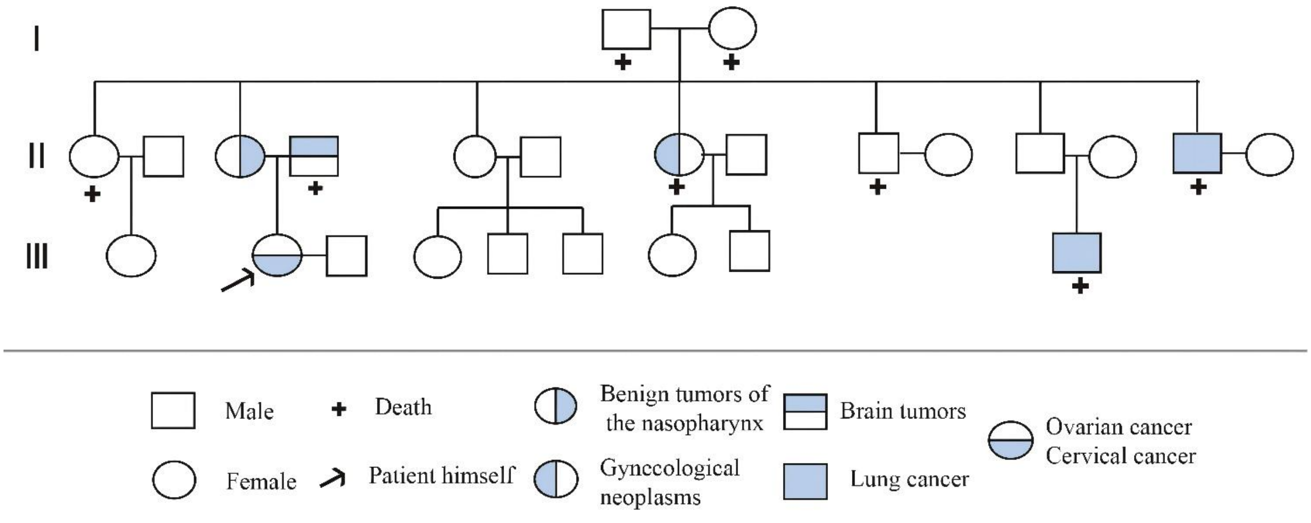

Supplement: Supplementary file 1 [file DataSheet_1.pdf]
